# The role of the cardiac lymphatic system in heart failure “reverse remodeling”: from developmental signals to druggable targets

**DOI:** 10.3389/fimmu.2026.1795814

**Published:** 2026-04-22

**Authors:** Tingxuan Huang, Teng Qi, Lingjun Yao, Zhentao Zhu, Chenyu Li, Pengxiang Tang, Zeyu Meng, Zheyu Wen, Tingyu Wang, Sui Liu, Peilin Xie, Zilin Li, Jing Hu

**Affiliations:** 1 School of Medicine, Northwest University, Xi’an, Shaanxi, China; 2 School of Medicine, The Chinese University of Hong Kong, Shenzhen, China; 3 Department of Cardiovascular Surgery, Xijing Hospital, Fourth Military Medical University, Xi’an, China

**Keywords:** cardiac lymphatic system, heart failure, lymphangiogenesis, myocardial fibrosis, reverse remodeling

## Abstract

Despite advances in therapies targeting hemodynamic and neurohormonal axes in heart failure (HF), incomplete reverse remodeling (RR) characterized by persistent myocardial edema and fibrosis remains a major clinical challenge. This review posits that dysfunction of the cardiac lymphatic system, a critical but understudied pathway for interstitial fluid and immune cell clearance, constitutes a fundamental barrier to complete myocardial recovery. We synthesize current evidence outlining the anatomy, developmental biology, and physiological role of cardiac lymphatics in maintaining myocardial fluid homeostasis and immune surveillance. In the context of HF, the lymphatic system undergoes a dynamic evolution: an initial compensatory lymphangiogenic response in the acute phase facilitates the clearance of edema and inflammatory cells, while its subsequent exhaustion or impairment in chronic HF perpetuates a vicious cycle of inflammation, fibrosis, and adverse remodeling. Central molecular pathways, including the VEGF-C/VEGFR-3 axis and transcriptional regulators like PROX1/FOXC2, govern lymphatic growth, integrity, and function. Furthermore, lymphatics actively modulate post-injury immune responses via specialized mechanisms such as CCL21/CCR7-guided cell trafficking. Therapeutically, augmenting cardiac lymphangiogenesis presents a promising strategy to enhance fluid drainage, resolve maladaptive inflammation, and directly support cardiomyocyte survival, thereby creating a conducive milieu for RR. However, translating this potential requires overcoming translational hurdles related to intervention timing, comorbidity-specific lymphatic dysfunction, and the development of targeted delivery systems. This review concludes that harnessing the cardiac lymphatic system represents a paradigm-shifting therapeutic avenue, complementary to existing regimens, with the potential to promote more complete and sustainable reverse remodeling in heart failure.

## 1 Introduction

Reverse remodeling (RR) is defined as the improvement or restoration of cardiac structure and function toward a more normal state, with left ventricular reverse remodeling (LVRR) being the primary clinical focus (1, 2). Following interventions such as pharmacotherapy, device therapy, surgery, or physiological processes like pregnancy, some patients exhibit sustained cardiac improvement, while others show incomplete recovery or even further deterioration in cardiac structure and function, a process termed adverse remodeling (AR) (3, 4).

Definitions of LVRR vary among studies. Traditional assessments rely predominantly on echocardiography and cardiac magnetic resonance (CMR). LVRR is commonly defined as an absolute increase in left ventricular ejection fraction (LVEF) of >10% or its normalization to ≥50%, accompanied by a >10% reduction in left ventricular dimensions (5–7). In evaluating LVRR, echocardiography can assess left ventricular filling pressure via the E/e′ ratio, which typically decreases as diastolic function improves during RR. Furthermore, CMR monitors a spectrum of left ventricular remodeling indices to quantify the extent of LVRR. During LVRR, CMR demonstrates a rapid and significant reduction in left ventricular mass (LVM), decreased maximal interventricular septal thickness (IVSmax), and improved global longitudinal strain (GLS), indicating enhanced myocardial contraction efficiency (8). Myocardial native T1, a known CMR imaging biomarker, directly quantifies myocardial fibrosis. Clinical studies have identified a native T1 value < 1073 ms as an independent predictor for post-procedural reductions in left ventricular end-diastolic volume (LVEDV) and end-systolic volume (LVESV), suggesting T1 mapping may be a valuable tool for predicting LVRR (9–11). CMR T2 mapping primarily reflects the degree of myocardial edema. In patients with aortic stenosis (AS) undergoing transcatheter aortic valve replacement (TAVR), those with pre-procedurally elevated T2 values (>70.2 ms), often associated with eccentric hypertrophy and reduced LVEF, showed significant decreases in T2 values during post-procedural LVRR, accompanied by improved LVEF and reduced LVEDV, indicating that T2 reduction can serve as evidence for LVRR (12, 13).

Additionally, clinical trials have established an association between N-terminal pro-B-type natriuretic peptide (NT-proBNP) levels and the degree of reverse remodeling as well as prognosis (14). NT-proBNP, encoded by the NPPB gene, is cleaved and further processed by proteases such as furin or corin. It has a relatively long half-life (120 minutes) in circulation, with levels normally very low but markedly elevated in heart failure (15–17). Compared to patients who did not reach the target, achieving an NT-proBNP level <1000 pg/ml within 12 months was associated with a significantly greater increase in EF (9.9 ± 8.8% vs. 2.9 ± 7.9%; p < 0.001) and lower LV volumes (14).

Current research on cardiac diseases predominantly focuses on hemodynamics, neuroendocrine activation, and inflammation. Regarding hemodynamics, given the reduced pump function and impaired filling in heart failure, traditional strategies include the long-term use of β-blockers to induce RR by reducing myocardial contractility and heart rate while improving metabolic efficiency (18, 19). However, in patients with heart failure with mildly reduced ejection fraction, β-blockers may paradoxically increase the risk of atrial fibrillation or death (20). Excessive activation of the sympathetic nervous system and the renin-angiotensin-aldosterone system contributes to myocardial dysfunction. Consequently, traditional approaches primarily employ angiotensin-converting enzyme (ACE) inhibitors to reduce angiotensin II and aldosterone production, promoting vasodilation and decreasing sodium and water retention (18, 21). Similarly, mineralocorticoid receptor antagonists (MRAs) counteract aldosterone’s effects by binding to its receptor (18). Nonetheless, these neurohormonal therapies exhibit a ceiling effect, where combination therapy or dose escalation does not continuously improve prognosis or achieve complete RR. Moreover, potent sympathetic inhibition may inadvertently increase mortality due to excessive suppression (22).

Furthermore, traditional research links the expression of various cytokines post-myocardial injury to cardiac RR. Clinically, patients with high levels of IL-13 and low levels of fibroblast growth factor-2 (FGF-2) exhibit a greater degree of RR and better outcomes following cardiac resynchronization therapy (CRT) (23). This suggests that certain factors (e.g., IL-13, IL-1α, IL-1β, IL-4) may promote RR, while others (e.g., FGF-2, IL-6, epidermal growth factor) may promote fibrosis and inhibit RR (23). Additionally, traditional studies indicate that elevated pro-inflammatory cytokines (e.g., IL-6, IL-8) in chronic heart failure are closely associated with adverse structural changes. An elevated ratio of matrix metalloproteinase-2 (MMP-2), which degrades the extracellular matrix, to its tissue inhibitor (TIMP-2) is a key mechanism driving adverse LV remodeling and functional deterioration (24). CRT can reverse this process through anti-inflammatory effects, ultimately inducing RR (25). However, anti-inflammatory therapies like CRT are not universally effective, and their efficacy highly depends on the patient’s pre-existing inflammatory and fibrotic status (23–25).

Despite the considerable success of therapies targeting hemodynamics, neuroendocrine pathways, and inflammation, these approaches have not enabled all patients to achieve complete cardiac RR. Many treated patients continue to exhibit persistent myocardial interstitial edema or fibrosis, along with incomplete RR, highlighting the urgent need for novel therapeutic avenues.

We hypothesize that the limitations of traditional therapies may stem from their inadequate addressing of the “fluid/immune clearance pathway.” In cardiovascular diseases, an imbalance between fluid filtration across capillary walls and its clearance from the myocardial interstitium via lymphatic capillaries, often due to altered capillary permeability, leads to excessive fluid accumulation within the myocardial interstitium or cells, resulting in myocardial edema (26, 27). Edema itself exacerbates pathology and often precedes irreversible changes in myocardial interstitial structure. Conversely, the accumulation of various cytokines, including TNF-α, TGF-β, and different interleukins, which activate inflammatory transcription factors like NF-κB, contributes to the pathogenesis of inflammatory cardiomyopathies. This buildup ultimately drives myocardial fibrosis and structural remodeling, with the effects of these cytokines often being concentration-dependent and dual-natured (28). Clinically, traditional methods aim to modify myocardial structure or reduce fluid production (e.g., diuretics for sodium and water excretion) but do not actively enhance tissue fluid drainage capacity. Regarding the clearance of accumulated cytokines, conventional anti-inflammatory therapies are often ineffective or cause significant side effects because they fail to fundamentally resolve the issue of immune clearance.

The cardiac lymphatic system potentially addresses this critical gap. It maintains myocardial fluid homeostasis through its specialized capillary absorption mechanism and a cardiac contraction-dependent transport system. Impairment of this system leads to myocardial edema and functional deterioration (29, 30). Moreover, the cardiac lymphatic system facilitates immune clearance. Under physiological conditions, LYVE1 expression is primarily confined to the initial and pre-collecting lymphatic vessels within the lymphatic system (31, 32). Lymphatic endothelial cells express the receptor LYVE-1, which interacts with hyaluronan on immune cell surfaces via CD44, mediating immune cell entry into lymphatic vessels (33–35). Additionally, lymphatic endothelial cells secrete the chemokine CCL21, which guides immune cell migration towards lymphatics via its receptor CCR7 (36). They also express adhesion molecules that promote immune cell adhesion and trans-endothelial migration into lymphatic vessels, thereby accelerating the clearance of immune cells and inflammatory mediators (33).

Therefore, enhancing cardiac lymphatic function may emerge as a novel therapeutic strategy. By actively improving fluid transport and immune waste clearance, it could address the shortcomings of traditional therapies and potentially promote more effective and complete cardiac RR.

## 2 Overview of the anatomy, physiology, and development of the cardiac lymphatic system

### 2.1 Anatomy of the cardiac lymphatic system

**Figure 1:**
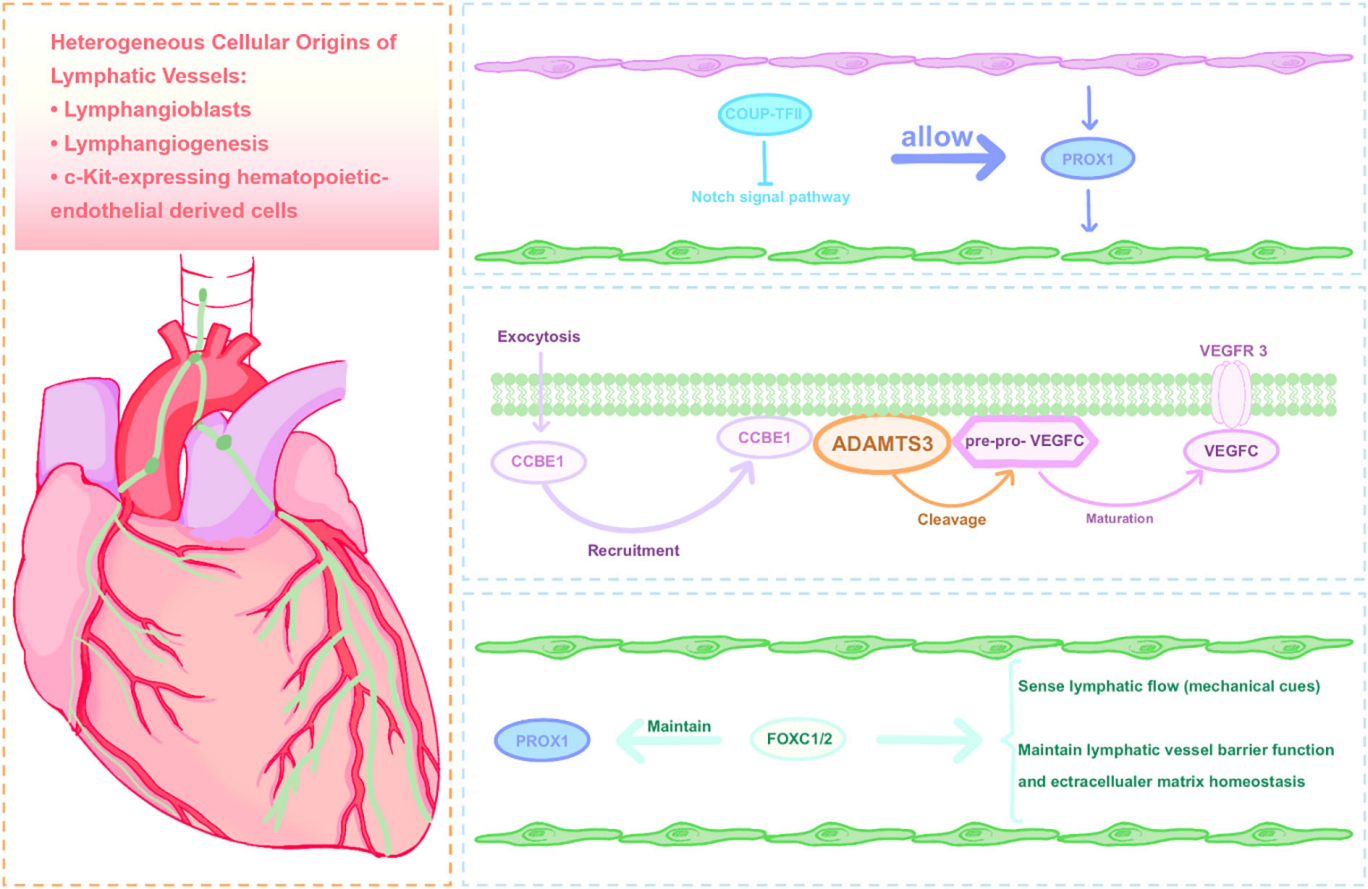
Key molecular mechanisms regulating cardiac lymphatic system development.

The cardiac lymphatic system is organized across three layers: the endocardium, myocardium, and epicardium, with a defined anatomical course (**Figure 1**). Cardiac lymph originates from the subendocardial plexus, which is particularly dense in the papillary muscles, and from the sparse lymphatic capillaries within the myocardial layer. These initial vessels converge to form larger lymphatics that drain lymph centrifugally towards the subepicardial layer (29, 37). The subepicardium contains valved pre-collecting lymphatic vessels. These vessels course along the anterior and lateral coronary arteries and posterior to the coronary sinus, extending from the cardiac apex toward the base, where they progressively merge into larger collecting lymphatics. Upon exiting the heart surface, these collecting vessels ultimately drain into mediastinal lymph nodes located below the aortic arch and around the trachea. From there, lymph returns to the venous circulation via the thoracic duct or right lymphatic trunk (37). Notably, the valved pre-collecting vessels in the subepicardium contain few lymphatic smooth muscle cells. This anatomical feature implies that lymph propulsion in the heart relies primarily on the mechanical forces generated by myocardial contraction and torsion, rather than on intrinsic lymphatic vessel contraction (29, 38).

It is important to distinguish the lymphatic system from the cardiovascular system. Unlike the closed, bidirectional blood circulation, the lymphatic system constitutes a unidirectional transport pathway from the extracellular space to the venous system. This unidirectionality is enabled by the structure of lymphatic capillaries, where endothelial cells overlap with “button-like” junctions that function as inwardly directed valves, permitting only the unidirectional inflow of interstitial fluid (39). These specialized structures work in concert with cardiac pulsation to maintain myocardial fluid balance and immune cell transport. They play a critical role in clearing edema and regulating inflammation during cardiac pathology.

### 2.2 Developmental signaling of the cardiac lymphatic system

#### 2.2.1 General molecular mechanisms of lymphatic development

The development of the cardiac lymphatic system is a complex, programmed process orchestrated by a sequence of key molecular signals. Current consensus holds that lymphatic endothelial cells (LECs) originate from embryonic veins (40, 41). During embryogenesis, a subset of venous endothelial cells, primarily in locations like the cardinal veins, begins to express the homeobox transcription factor PROX1. This expression event signifies the specification of these venous endothelial cells into the lymphatic endothelial lineage (40). Additional factors, including the nuclear hormone receptor COUP-TFII, as well as Notch and retinoic acid signaling pathways, are crucial for the precise regulation of PROX1 expression and the initial specification of LECs (41).

Following LEC specification, the VEGFC-VEGFR3 axis constitutes the core growth factor signaling pathway for lymphatic development. VEGFC, signaling selectively through its receptor VEGFR3 on LECs, activates downstream pathways that drive LEC migration, proliferation, and the construction of the lymphatic network (42). Notably, VEGFC can also bind to VEGFR2 (43, 44), and the expression of VEGFR3 is not exclusive to lymphatic endothelial cells (45, 46). However, the unprocessed VEGF-C precursor exhibits weak activity, and its complete activation depends on proteolytic cleavage by the metalloproteinase ADAMTS3. This process is critically facilitated by the scaffold protein CCBE1, which coordinates the assembly of the activation complex on the surface of lymphatic endothelial cells (43–47).

#### 2.2.2 Organ-specific aspects of lymphatic development in the heart

The origin of lymphatic endothelial cells has been a classic debate in the field of lymphology for over a century. In 1902, Sabin proposed that lymphatic vessels originate from the budding of embryonic veins (48); however, in 1910, Huntington and McClure suggested that lymphatic vessels could differentiate *in situ* within the mesenchyme, forming independently from veins before later establishing connections with the vascular system (49). For a long time, the venous origin theory has dominated research on systemic lymphatic development in mammals. Nevertheless, recent studies indicate that organ specificity may be key to understanding this debate, with the development of cardiac lymphatic vessels being particularly illustrative (50, 51).

In the specific context of the heart, the cellular origin of lymphatic vessels exhibits unique heterogeneity. Klotz et al., using lineage tracing techniques, were the first to demonstrate (50) that the formation of the cardiac lymphatic network is, at least in part, independent of venous budding, suggesting the existence of non-venous derived lymphatic endothelial progenitors. Utilizing lineage tracing tools such as Tie2-Cre and Vav1-Cre, this study identified that a subset of cardiac lymphatic endothelial cells (LECs) originates from hemogenic endothelium, providing modern molecular evidence for the “mesenchymal origin” theory proposed by early scholars like Huntington. Additionally, Stanczuk, using cKit as a marker, demonstrated that mesenteric lymphatic vessels might also originate from hemogenic endothelium (52), collectively pointing to hemogenic endothelium as a potentially common source of lymphatic precursors. Further research revealed that in the developing heart, approximately 19% of lymphatic vessels were YFP-negative (i.e., non-venous origin), while 78% were YFP-positive (venous origin), confirming the mixed origin of cardiac lymphatic endothelium (50).

In the context of cardiac valves, the transcription factors FOXC1/2 play a critical role (53, 54): they maintain appropriate PROX1 expression levels in specific valve endothelial cells and are involved in sensing mechanical signals generated by lymphatic flow, thereby regulating LEC gene expression and structural remodeling.

In recent years, the organ-specific characteristics of cardiac lymphatic development have been further elucidated. Regarding whether the epicardium contributes to cardiac lymphatic vessels, Wilting et al., through chick-quail chimera transplantation experiments, demonstrated that the epicardium can provide hemangioblasts but not lymphangioblasts (55). However, the latest research reveals that cardiac LECs may originate from multiple progenitor populations, including venous endothelium and Isl1+ precursor cells derived from the second heart field (SHF) (56). Furthermore, the cardiac lymphatic system promotes tissue repair after myocardial injury by regulating immune cell clearance and inflammation resolution, a function particularly prominent in the adult heart. Following myocardial infarction, stimulating lymphangiogenesis facilitates immune cell clearance via a LYVE-1-dependent mechanism (57), reducing inflammation, fibrosis, and pathological remodeling, thereby improving cardiac function. These data suggest that lymphangiogenesis may represent a therapeutic target for promoting repair after cardiac injury.

### 2.3 Impact of aging and comorbidities on cardiac lymphatic structure and function

#### 2.3.1 Impact of aging

With advancing age, the lymphatic system, particularly lymph nodes, undergoes significant structural alterations. Lymph nodes become fibrotic overall, with increased collagen deposition invading T-cell zones and a general disorganization of architecture, blurring the demarcation between T- and B-cell areas (47, 58–60). At the cellular level, in mesenteric lymph nodes, fibroblastic reticular cells (FRCs) decrease in number. The proportion of immature FRC subsets (VCAM-1lo) declines, accompanied by reduced expression of the lymphotoxin-beta receptor (LTBR) and the stem cell marker SCA-1, indicating impaired differentiation and maintenance potential (58, 61). Concurrently, the reticular network formed by FRCs becomes denser and more disordered (59). The area occupied by follicular dendritic cells (FDCs) diminishes, compromising germinal center formation and antibody production (62). A decline in lymphatic endothelial cell function, manifested as reduced VEGFR-3 signaling and increased apoptosis, leads to enhanced lymphatic permeability and impaired antigen and cell transport, accompanied by a decrease in lymphatic vessel density and network complexity (63, 64). Notably, the age-related reduction in lymphatic contractile function is primarily attributed to a decline in the pumping function of lymphatic smooth muscle cells, rather than endothelial cell mechanisms (65–67).High endothelial venules (HEVs) undergo morphological changes that hinder lymphocyte homing. Molecularly, expression of the T-cell homeostasis chemokine CCL21 and the key developmental signal LTBR is reduced (58, 59). Collectively, these structural and microenvironmental decays result in hindered naive T-cell migration within nodes, insufficient survival signaling, and weakened efficiency of antigen presentation and lymphocyte coordination (68). They form an important structural basis for the diminished immune response characteristic of aging.

#### 2.3.2 Impact of comorbidities

Following myocardial infarction (MI), the cardiac lymphatic system undergoes a dynamic evolution from acute compensation to chronic failure. In the acute phase, compensatory lymphangiogenesis is primarily driven via the VEGF-C/VEGFR-3 signaling axis to clear edema and inflammatory cells (69–71). However, as the condition progresses into the chronic phase, lymphangiogenesis wanes, lymphatic structures degenerate, and inflammation promotes a transition in endothelial junctions from the permeable “button-like” phenotype to a closed “zipper-like” conformation. This transition severely impairs lymphatic drainage function (69, 72, 73). Further studies indicate that downregulated EphrinB2 expression in the infarct zone fails to promote VEGFR3 expression and lymphangiogenesis via activation of the CDK5/ISL1 pathway (74, 75). Moreover, infiltrating CD4^+^/CD8^+^ T cells secreting IFN-γ further inhibit lymphangiogenesis (69). These combined defects lead to the persistent accumulation of tissue fluid, inflammatory cells, and mediators, establishing a vicious cycle of chronic edema and inflammation. Notably, beyond their drainage role, LECs can actively secrete signaling molecules such as Reelin. These molecules support cardiomyocyte survival and proliferation directly, for instance by activating the PI3K/AKT pathway, thereby reducing apoptosis and fibrosis (76–78). Consequently, lymphatic dysfunction ultimately exacerbates myocardial fibrosis and adverse cardiac remodeling, driving heart failure progression. Given these multifaceted mechanisms, modulating lymphangiogenesis and endothelial function(such as VEGF-C, EphrinB2, ADM, and Apelin) has emerged as an important potential strategy for promoting post-MI cardiac repair (76). In recent years, methodological innovations in the therapeutic application of Apelin for myocardial infarction have primarily focused on overcoming its short half-life (5–8 minutes) and systemic side effects—issues that previously hindered its clinical translation (79). Tang et al. developed a microparticle-mediated delivery system, which involved fabricating PLGA nanofibers via coaxial electrospinning, followed by cryogenic grinding to generate apelin-loaded microparticles. These were then embedded within a fibrin-thrombin gel patch (80). This epicardial patch enabled sustained release of apelin, with 62% released within the first 7 days and approximately 84% by day 28 in both acute and chronic phase mouse models of myocardial infarction. This delivery system consequently reduced cardiomyocyte apoptosis, enhanced angiogenesis, and inhibited cardiac fibroblast activation by suppressing the TGF-β/Smad2/3 signaling pathway (80). Complementing this approach, researchers have also designed enzyme-responsive self-assembled multivalent apelin ligands (SAMPs), which achieve targeted accumulation at the myocardial infarction site by responding to locally upregulated matrix metalloproteinases, thereby prolonging retention time and enhancing receptor binding affinity (81). Collectively, these advances in targeted and sustained delivery strategies position apelin as a promising therapeutic candidate for cardiac repair following myocardial infarction. Furthermore, ADM exerts certain direct protective effects on myocardial infarction. Regarding its direct protective role, research by Hinrichs et al. demonstrated that both ADM and its precursor, ProADM, significantly alleviate myocardial ischemic injury (82). Treating ischemic cardiomyocytes with either ProADM or ADM markedly increased cell survival rates and reduced caspase 3/7 activity, thereby inhibiting apoptosis (82). Notably, ProADM and ADM exhibit functional differences in inflammation regulation: ProADM can induce pro-inflammatory cytokine expression to promote local inflammation, while also suppressing excessive leukocyte activation. In contrast, ADM primarily functions by reducing chemokine expression, exerting an anti-inflammatory effect (82).

Atherosclerosis impairs the structure and function of the aortic lymphatic system through multiple mechanisms. A central mechanism involves direct attack by inflammatory cytokines, such as TNF-α, which inhibit LEC proliferation, migration, and lymphangiogenic capacity, leading to structural damage. This process is accompanied by the downregulation and diminished nuclear translocation capability of the key transcription factor FOXC2, weakening its normal regulatory control over LECs. Simultaneously, TNF-α binds to its downstream signaling molecule TRAF2, activating pro-inflammatory pathways that further exacerbate cellular injury. Importantly, the loss or functional inhibition of FOXC2 potentiates this detrimental TNF-α–TRAF2 interaction. Furthermore, expression of proteins crucial for lymphatic valve formation, including Laminin α5 and Integrin α9, is reduced, impairing the rhythmic contraction and unidirectional drainage function of lymphatics. Ultimately, these molecular and cellular pathologies converge to cause severe dysfunction in the drainage capacity of adventitial lymphatics. This results in the accumulation of inflammatory mediators, lipids, and immune cells within the vessel wall, creating a vicious cycle that accelerates atherosclerotic progression (83, 84).

In addition to these inflammatory mechanisms, the lymphatic system plays a crucial role in reverse cholesterol transport, a process that shuttles excess cholesterol from peripheral tissues to the liver for elimination (85). Lymphatic vessels within the arterial wall directly participate in reverse cholesterol transport, and their dysfunction impairs cholesterol clearance, thereby promoting lipid accumulation within plaques (85). Studies in mouse models of hypercholesterolemia have revealed multifaceted lymphatic dysfunction, including reduced contractility of collecting lymphatic vessels, impaired valvular function, and increased vascular permeability, affecting both lymphatic endothelial cells and smooth muscle cells (86). These findings suggest that therapeutic strategies targeting lymphatic function could enhance reverse cholesterol transport and mitigate atherosclerosis progression.

Diabetes mellitus also significantly affects the function of the cardiac lymphatic system. In experimental models of diabetes, characteristic pathological alterations are observed in the lymphatic system. Oxidative stress within the lymph is markedly enhanced, evidenced by a sharp increase in concentrations of lipid peroxidation products (e.g., diene conjugates and malondialdehyde) and a concomitant depletion of endogenous antioxidant defenses (e.g., reduced glutathione). This state of oxidative imbalance is closely linked to a coagulation dysfunction within the lymph, manifesting as shortened clotting time, increased release of endothelial function markers, and inhibited fibrinolytic activity. This creates a state of “lymphatic hypercoagulability,” which in turn leads to severe impairment of cardiac lymphatic drainage function (87).

In a mouse model of type 2 diabetes (db/db mice), the contraction amplitude, frequency, and pumping flow of collecting lymphatic vessels are significantly reduced (88). Concurrently, lymphatic permeability in diabetic vessels increases over 130-fold, attributed to impaired nitric oxide signaling and subsequent activation of phosphodiesterase 3, a defect that can be reversed by L-arginine supplementation (89). These findings indicate that diabetes induces lymphatic contractile dysfunction via KATP channel-mediated mechanisms and lymphatic barrier disruption through NO/PDE3-mediated pathways (88, 89).

In addition to functional deficits, genetic studies have linked lymphatic regulatory factors to susceptibility to type 2 diabetes. Genome-wide association studies have consistently identified PROX1 as a susceptibility locus across multiple ethnic populations (90). The PROX1 rs340874 variant is significantly associated with type 2 diabetes in both European and Chinese populations, with the risk allele correlated with elevated 2-hour glucose levels during OGTT and impaired insulin secretion (90). Furthermore, GWAS catalog entry rs114526150 shows significant association with type 2 diabetes in large European cohorts (P = 4 × 10^−7^). In contrast, FOXC2 variants are not directly associated with diabetes risk but correlate with metabolic phenotypes such as elevated triglycerides and C-peptide levels (91). FOXC2 C512T and G350T polymorphisms are associated with insulin resistance, though not with diabetes risk itself (92).

Collectively, these studies demonstrate that type 2 diabetes induces significant lymphatic dysfunction—reduced pumping capacity and increased permeability—potentially exacerbating tissue fluid accumulation and immune dysregulation. Genetic evidence implicates PROX1 as a shared link between lymphatic biology and metabolic disease (90).

## 3 Alterations of the lymphatic system in heart failure and reverse remodeling

### 3.1 Changes in the lymphatic system during the acute phase of heart failure

**Figure 2:**
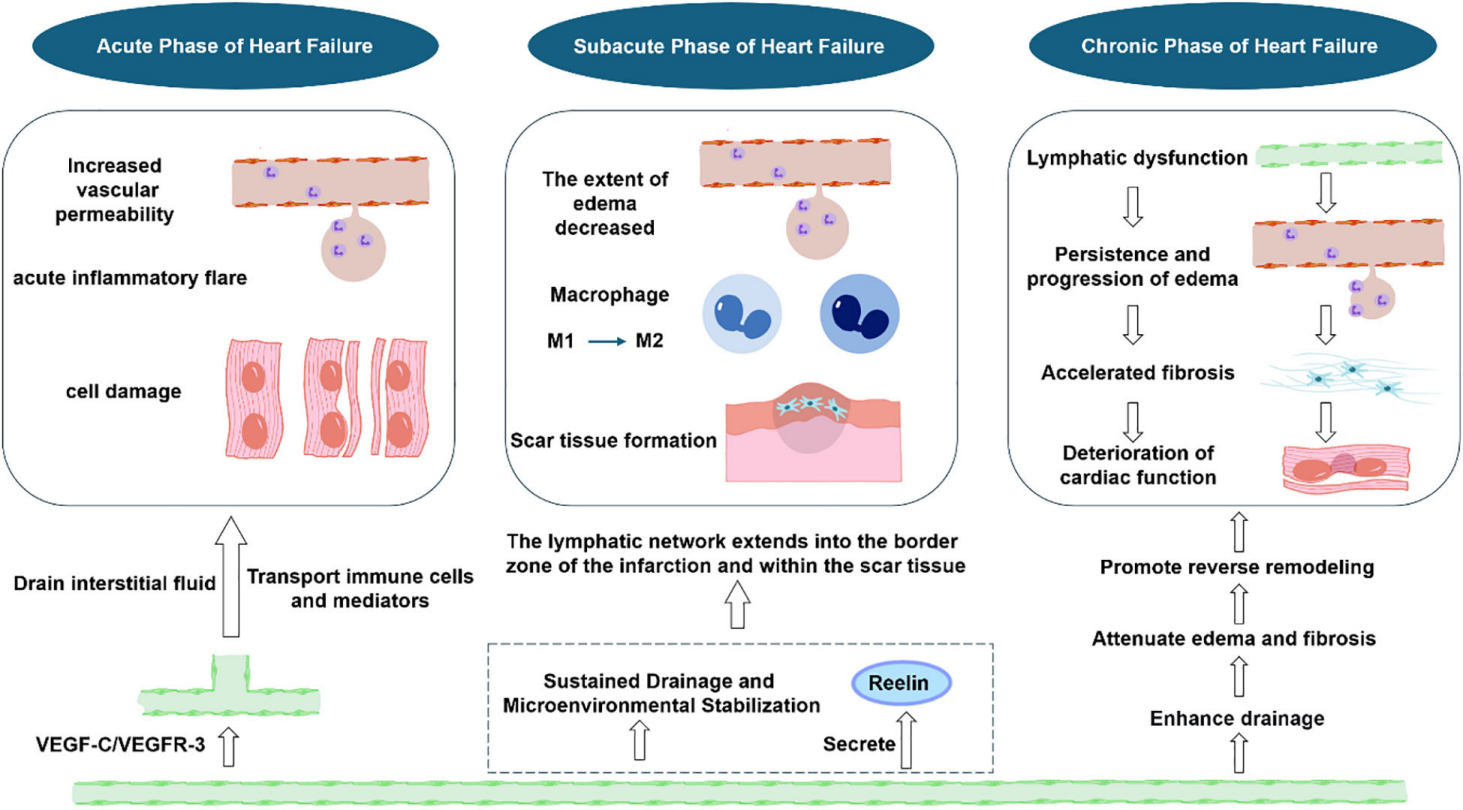
Temporal dynamics of the cardiac lymphatic system across the progression of heart failure.

Following acute myocardial infarction in the setting of heart failure, ischemic injury induces increased microvascular permeability (**Figure 2**). This leads to the extravasation of protein-rich fluid into the myocardial interstitium, resulting in acute myocardial edema (93, 94). Concurrently, a robust inflammatory response is triggered (94–96), characterized by the infiltration of numerous immune cells and the release of pro-inflammatory cytokines (97–100). These processes collectively contribute to the rapid deterioration of cardiac systolic function and accelerate cardiac remodeling (50, 101, 102). During this phase, the heart initiates a compensatory lymphangiogenic response (69, 103), primarily mediated through the VEGF-C and VEGF-D signaling axis and their receptor VEGFR-3 (69, 102, 104). Functioning as an efficient drainage network, the cardiac lymphatic system actively clears accumulated interstitial fluid (99), directly alleviating myocardial edema and reducing ventricular wall stiffness. Furthermore, lymphatic vessels serve as a “conduit for inflammatory clearance,” (70) transporting immune cells such as neutrophils and macrophages, along with their inflammatory mediators, away from the heart to regional draining lymph nodes (94). This process effectively promotes the resolution of local inflammation and prevents its progression into a chronic, detrimental state (69, 103). In addition to this passive clearance function, emerging evidence suggests that VEGF-C and VEGF-D may exert direct anti-inflammatory effects. VEGF-C signaling via VEGFR-3 has been shown to regulate inflammatory cell recruitment and cytokine production independently of its lymphangiogenic functions (105–107).Through this dual mechanism of managing fluid overload and modulating the immune response, the lymphatic system curbs the upstream drivers of myocardial fibrosis and adverse remodeling, thereby fostering a favorable microenvironment for cardiac functional recovery (74, 103, 108). Research indicates that therapeutically enhancing cardiac lymphangiogenesis via signaling pathways such as VEGF-C/VEGFR-3 can further amplify these protective effects (102, 109). This leads to significant improvements in myocardial fluid balance, reductions in inflammation and fibrosis, and ultimately, promotes the recovery of systolic function and facilitates left ventricular reverse remodeling (LVRR). Mechanistically, enhanced lymphatic function promotes reverse remodeling by alleviating myocardial edema (thereby reducing ventricular wall stress and improving compliance) and resolving inflammation (thereby inhibiting profibrotic signaling and extracellular matrix deposition) (110). Consequently, a functionally intact lymphatic system is a crucial intrinsic safeguard for the transition from acute injury to stable repair following heart failure (103, 111).

### 3.2 Changes in the lymphatic system during the subacute phase of heart failure

As the heart progresses into the subacute phase of post-infarction repair and scar formation (27, 112), the ongoing processes of lymphatic system growth and pathway re-establishment play a supportive role in facilitating LVRR (50, 69, 76). Lymphatic endothelial cells (LECs) are capable of secreting Reelin (113), an extracellular matrix signaling molecule. In models of myocardial injury, LEC-derived Reelin acts directly on cardiomyocytes, primarily via the integrin-β1 signaling pathway, to promote their survival and proliferation while inhibiting apoptosis (71, 114, 115). Therapeutic supplementation of Reelin has been demonstrated to significantly reduce scar size and improve cardiac function (94, 103). Moreover, newly formed lymphatic vessels are observed not only in the peri-infarct border zone but also within the developing scar tissue itself. Histopathological evidence suggests that these intrascar lymphatics may participate in the maturation of fibrotic tissue and the final remodeling of the scar (69, 116, 117), potentially by aiding in the drainage of excess protein and fluid (118, 119), thereby influencing scar stability and architecture (103, 120).

Despite these advances, important unanswered questions remain regarding the mechanisms by which Reelin production is regulated in LECs. The upstream signals controlling Reelin expression and secretion in cardiac lymphatic endothelial cells are not fully understood. Recent proximity-dependent proteomic studies have identified the adhesion receptor vascular endothelial cadherin as playing a necessary role in controlling Reelin secretion by LECs, both *in vitro* and *in vivo* (121). However, whether mechanical stimuli such as fluid shear stress, inflammatory signals, or intercellular communication with surrounding cell types regulate LEC-derived Reelin in the injured heart remains to be elucidated. Furthermore, the relative contributions of Reelin produced by different cellular sources—LECs versus other cell types such as cardiac Schwann cells (115)—to cardiac repair warrant further investigation.

Transitioning from the organized repair of the subacute phase, the long-term adaptation of the lymphatic system becomes critical as heart failure enters a chronic state.

### 3.3 Changes in the lymphatic system during the chronic phase of heart failure

In the chronic stage of heart failure, the endogenous lymphangiogenic response is often attenuated or exhausted, leading to impaired lymphatic drainage function. This impairment results in a vicious cycle: persistent myocardial edema exacerbates fibrosis (122, 123), which in turn further compromises cardiac function. Importantly, however, experimental evidence suggests that this detrimental cycle is not irreversible (124, 125). Therapeutic interventions aimed at stimulating lymphangiogenesis and enhancing lymphatic function—such as targeted delivery of VEGF-C or administration of VEGFR-3 agonists (126)—even at this chronic stage (127–129), can significantly improve lymphatic drainage (130, 131). This improvement alleviates myocardial edema and fibrosis, leading to partial reversal of cardiac dilation and dysfunction, thereby breaking the self-perpetuating cycle of deterioration.

### 3.4 The association between the lymphatic system and cardiac reverse remodeling

The collective evidence underscores a direct link between a functional lymphatic network and the promotion of reverse cardiac remodeling (108, 132, 133). Experimentally, the induction of lymphangiogenesis has been shown to significantly mitigate cardiac inflammation and fibrosis (116, 120), while continuously removing immune cells like macrophages to attenuate persistent inflammatory responses (134, 135). This creates a conducive environment for myocardial repair (69, 70). Furthermore, the modulation of lymphangiogenesis through key signaling pathways, notably VEGFR-3 (109), serves as a protective mechanism against pressure overload-induced cardiac dysfunction (103, 108, 135). In summary, across the acute, subacute, and chronic phases of heart failure, the lymphatic system exerts multifaceted protective effects by regulating fluid homeostasis, orchestrating immune responses, and directly influencing cardiomyocyte and scar biology (136). Targeted therapeutic strategies to enhance cardiac lymphatic function therefore represent a promising avenue for supporting myocardial recovery and achieving sustainable reverse remodeling (70, 116).

### 3.5 Changes in the lymphatic system in pressure overload, cardiac hypertrophy, and HFpEF

While myocardial infarction represents a major driver of heart failure, a significant and growing proportion of patients, particularly those with heart failure with preserved ejection fraction (HFpEF), develop the syndrome through non-ischemic pathways such as chronic pressure overload. Emerging evidence indicates that lymphatic dysfunction is not merely a consequence of ischemic injury but is intricately involved in the pathophysiology of pressure overload-induced cardiac hypertrophy and HFpEF (109, 132).

In models of chronic pressure overload induced by transverse aortic constriction (TAC), the cardiac lymphatic system undergoes a complex and often inadequate remodeling process. Studies have revealed that despite an initial compensatory lymphangiogenic response, this expansion is frequently insufficient to meet the increased demands for fluid and immune cell clearance. Using single-cell analyses in murine HF models, Heron et al. demonstrated that while TAC leads to an expansion of lymphatic capillaries, it simultaneously causes a loss of lymphatic valves and dysregulation of lymphatic barrier function (132, 137). This valve loss and junctional impairment critically undermine the unidirectional drainage capacity of the network, leading to persistent myocardial edema despite the presence of more lymphatic vessels. Functional studies inhibiting VEGFR-3 signaling after TAC have confirmed that compromised lymphangiogenesis exacerbates cardiac inflammation, particularly myeloid cell accumulation, and accelerates the development of left ventricular dilation and dysfunction, underscoring the protective role of an intact lymphatic system in non-ischemic HF (109).

The relevance of these findings is particularly pronounced in the context of HFpEF, a syndrome characterized by diastolic dysfunction, myocardial stiffening, and a high burden of comorbidities like hypertension, obesity, and type 2 diabetes. Cuijpers et al. have reviewed the evidence linking microvascular and lymphatic dysfunction to the key pathological steps in HFpEF, proposing that impaired lymphatic drainage contributes to a vicious cycle of chronic low-grade inflammation, myocardial edema, and fibrosis (131). Direct experimental evidence for this was recently provided by studies in a two-hit murine HFpEF model (high-fat diet plus L-NAME). These studies demonstrated that HFpEF progression is associated with significant cardiac lymphatic rarefaction (138). Crucially, inhibition of lymphangiogenesis in this model aggravated cardiac remodeling and dysfunction. Conversely, therapeutic lymphangiogenesis, achieved through the implantation of VEGF-C-secreting cardiac fibroblasts, ameliorated cardiac inflammation and fibrosis, leading to improved diastolic function (138). This provides proof-of-concept that enhancing lymphatic function could be a viable therapeutic strategy for HFpEF.

Furthermore, the concept that lymphatic insufficiency can directly cause diastolic dysfunction has been substantiated by elegant surgical models. Pu et al. established a mouse model of cardiac lymphatic dysfunction by ablating the main cardiac lymphatic collector vessels in an otherwise healthy heart. This isolated lymphatic impairment was sufficient to induce myocardial edema, inflammation, and fibrosis, culminating in significant cardiac hypertrophy and diastolic dysfunction (122). This work demonstrates that lymphatic failure is not merely a bystander but can be a primary driver of the morphological and functional changes characteristic of HFpEF.

In summary, across the spectrum of pressure overload and HFpEF, the cardiac lymphatic system emerges as a critical determinant of disease progression. Its dysfunction, characterized by insufficient lymphangiogenesis, valve loss, and impaired barrier function, actively contributes to the maladaptive inflammation, fibrosis, and diastolic stiffening that define these conditions. Therapeutic strategies aimed at restoring lymphatic health therefore hold substantial promise for a broad range of heart failure patients beyond those with ischemic etiology (139).

## 4 Mechanisms and molecular pathways

### 4.1 VEGF-C/VEGFR-3 and its regulation

**Figure 3:**
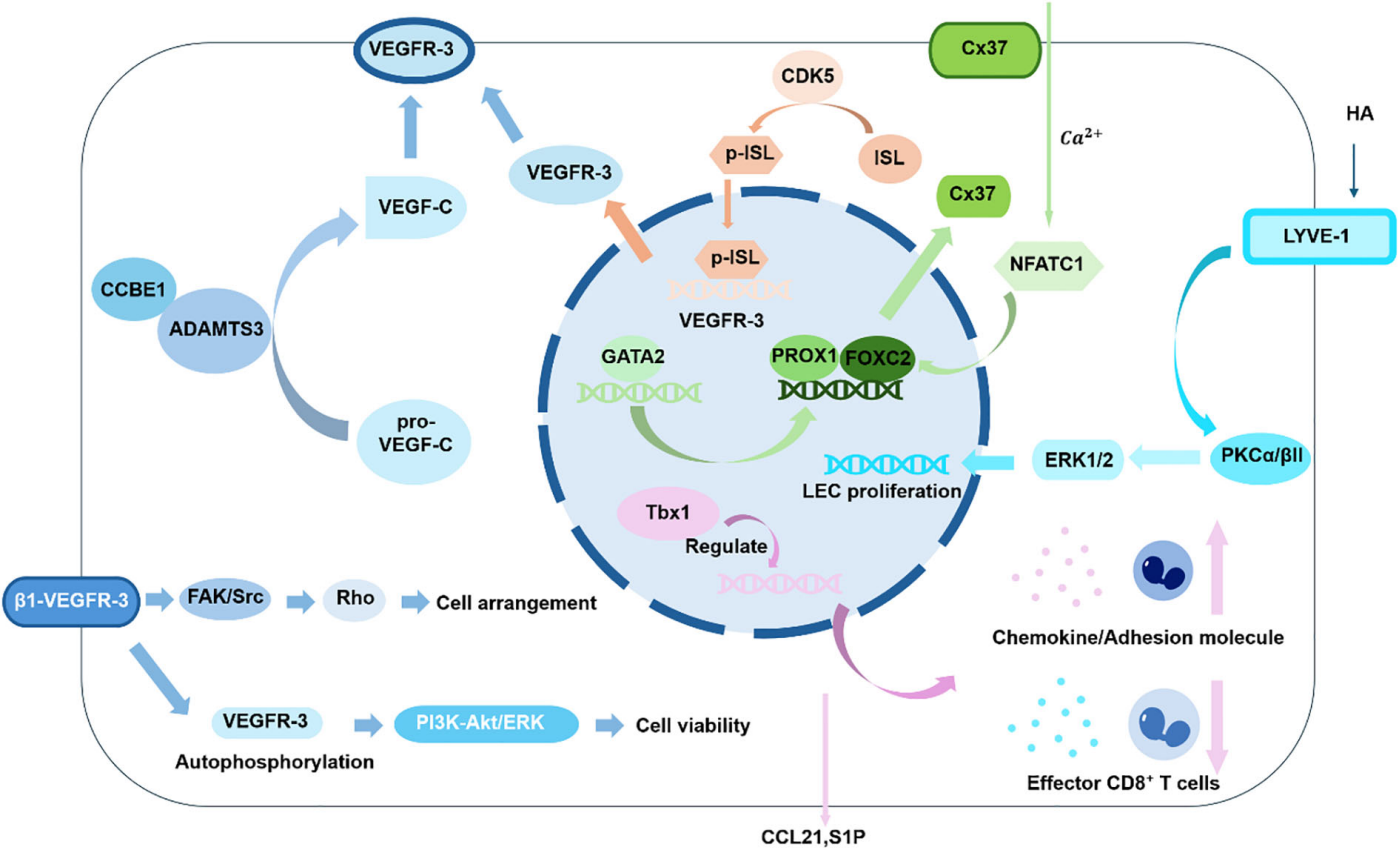
Schematic of the cardiac lymphatic regulatory network and its therapeutic targets.

The structure and function of the cardiac lymphatic system are under precise molecular control (**Figure 3**), with the VEGF-C/VEGFR-3 signaling axis serving as the central pathway mediating lymphangiogenesis and remodeling (140–142). In a Coxsackievirus B3 (CVB3)-induced viral myocarditis model, inflammatory stimuli promote macrophage infiltration and subsequent robust expression and secretion of VEGF-C (46). This growth factor acts in a paracrine manner on VEGFR-3 (143), which is highly expressed on the surface of cardiac lymphatic endothelial cells (LECs). Ligand binding induces receptor dimerization and autophosphorylation of the intracellular tyrosine kinase domains, thereby activating key downstream signaling pathways such as PI3K-Akt and ERK1/2. These signaling cascades significantly promote cardiac lymphangiogenesis by regulating LEC proliferation, migration, and lumen formation (128, 144, 145). Furthermore, the VEGF-C/VEGFR-3 pathway can mitigate the inflammatory response by inhibiting the p38 and JNK branches of the MAPK pathway (including phosphorylation of p38 and JNK), in a manner that does not affect ERK1/2 activation mediated by VEGFR-3 homodimerization (146, 147).

This action reduces the retention of Ly6G^+^ neutrophils within the myocardium and downregulates the expression of pro-inflammatory cytokines like IL-6, TNF-α, and IL-1β, ultimately leading to improved cardiac functional parameters (148, 149).

Building upon this foundational understanding, recent studies have revealed additional regulatory layers under hypoxic conditions. In this context, EphrinB2 promotes the expression of CDK5 and its interaction with ISL1 (150). This interaction induces CDK5-dependent phosphorylation of ISL1, facilitating its nuclear translocation. Once in the nucleus, ISL1 binds to the promoter region of VEGFR3, enhancing its transcriptional activity and upregulating VEGFR-3 expression. This CDK5/ISL1/VEGFR-3 signaling axis ultimately promotes LEC proliferation, migration, and lymphatic network formation. By enhancing lymphatic drainage, it accelerates the clearance of inflammatory cells after myocardial infarction, thereby improving cardiac repair and function (149).

It is important to note that VEGF-C requires a processing step for activation. The secreted pro-VEGF-C is initially inactive. Its maturation and activation depend on a regulatory network involving CCBE1 and ADAMTS3 (47). The specific mechanism involves CCBE1 (151), which, via its C-terminal collagen-like domain, specifically binds and recruits pro-VEGF-C. Simultaneously, through its N-terminal EGF-like domains, CCBE1 binds and precisely localizes ADAMTS3, also enhancing the protease’s activity when its concentration is suboptimal (45). The recruited ADAMTS3 then performs specific cleavage on pro-VEGF-C (152). The resulting mature VEGF-C gains high affinity for VEGFR-3, enabling effective binding and receptor activation. This initiates downstream signaling that drives LEC proliferation, migration, and survival, culminating in the formation of a functional lymphatic network (153).

Research further reveals complexity within this pathway. The highly homologous proteases ADAMTS2 and ADAMTS14 can also process pro-VEGF-C *in vitro*. Other proteases, such as plasmin, may cleave VEGF-C under specific pathological conditions; however, the characteristics of their cleavage products differ from those generated by physiological processing (154, 155).

### 4.2 Transcriptional and structural programming

Within the cardiac lymphatic system, the transcription factors PROX1, FOXC2, and GATA2 collectively regulate LEC identity and valve formation (156). Acting as an upstream hub, GATA2 directly responds to mechanical stimuli such as oscillatory shear stress (OSS) and physically interacts with Hdac3. Hdac3 recruits the histone acetyltransferase Ep300 to form an enhanceosome complex, which further promotes Gata2 expression (157), in turn activating and enhancing the expression of PROX1 and FOXC2 to initiate the valvulogenic program (158). PROX1 and FOXC2 function synergistically in a dual manner (159, 160). Firstly, within cardiac valve endothelium (161), they establish a PROX1-FOXC2-PDGF-B signaling axis that directly represses PDGF-B expression (54, 162). This repression is crucial for maintaining valve extracellular matrix (ECM) homeostasis and preventing pathological myxomatous degeneration (163). Secondly, at sites of lymphatic valve formation, PROX1 and FOXC2 integrate sustained laminar shear stress signals to cooperatively transcriptionally upregulate the expression of connexin 37 (Cx37) (164, 165). Gap junctions formed by Cx37 mediate local calcium influx, activating calcineurin (166). This leads to the dephosphorylation and nuclear translocation of the transcription factor NFATC1. Within the nucleus, NFATC1, along with PROX1, FOXC2, and GATA2, binds to an evolutionarily conserved Prox1 gene enhancer (167). This interaction forms a positive feedback regulatory loop. This loop not only stabilizes and amplifies PROX1 expression to cement LEC identity and suppress transdifferentiation towards hematopoietic lineages but also, through regulation of downstream targets like Cx37 (168, 169), orchestrates the alignment of valve endothelial cells, ECM remodeling, and the final morphogenesis of valve leaflets.

### 4.3 Stromal and glycocalyx interface

The stromal-glycocalyx interface and mechanotransduction mechanisms within the cardiac lymphatic system constitute a multidimensional regulatory network. The LYVE-1-hyaluronan (HA) axis mediates critical chemosignaling (170, 171): hyaluronan in the extracellular matrix binds to the LYVE-1 receptor on LEC surfaces, activating the downstream PKCα/βII-ERK1/2 signaling pathway (35, 172). This activation directly drives LEC proliferation, migration, and tubulogenesis, thereby promoting physiological lymphangiogenesis. Under pathological conditions, such as pressure overload, distinct cardiac macrophage subsets differentially utilize LYVE-1 for regulation. LYVE-1^+^ macrophages secrete pro-lymphangiogenic factors to maintain lymphatic network homeostasis. In contrast, LYVE-1^−^ macrophages express high levels of matrix metalloproteinases like MMP12, directly degrading the ECM and downregulating LYVE-1 surface expression on LECs, thereby driving pathological remodeling (126, 173, 174). During the inflammation resolution phase post-myocardial infarction, the LYVE-1 pathway is essential for clearing inflammatory cells (175); its impairment leads to chronic inflammation and worsened cardiac function (70, 176, 177).

Concurrently, interstitial flow acts as a key biophysical stimulus, influencing LEC-E CM interactions via two primary mechanisms (178). First, the accumulation of interstitial fluid causes ECM swelling, which exerts tension on anchoring filaments. This tension mechanically opens junctions between LECs, facilitating fluid uptake (179). Second, LECs sense mechanical cues from ECM stiffness, cell stretch due to fluid pressure, and flow-derived shear stress. Through mechanosensors such as the β1 integrin-VEGFR-3 complex, these mechanical signals are transduced into intracellular biochemical signals (180). This transduction regulates LEC proliferation, alignment, and valve formation, and feeds back to modulate ECM remodeling via integrin-mediated pathways (181, 182).

### 4.4 The immune trafficking axis

Following myocardial infarction, the cardiac lymphatic system actively modulates the local immune microenvironment through specific molecular and cellular mechanisms. At the level of cell guidance and entry, lymphatic endothelial cells employ spatially targeted exocytosis to release the chemokine CCL21 specifically at multicellular junctions (183–185). This localized release guides CCR7-expressing dendritic cells (DCs) to traverse the endothelium and enter the lymphatic vessels. Simultaneously, a sphingosine-1-phosphate (S1P) concentration gradient between the tissue and lymph, with higher concentrations in the lymph, provides a directional cue for cell migration towards draining lymph nodes (186, 187). The S1P receptor 1 (S1PR1) signaling pathway is particularly active within lymphatic collecting vessels, facilitating this transit (188).

In addition to CCL21, other molecular players orchestrate immune cell trafficking. Lymphatic endothelial hyaluronan receptor 1 (LYVE-1), highly expressed on initial lymphatic vessels, mediates the anchoring and transcellular migration of hyaluronan-coated immune cells, including macrophages and dendritic cells (70). This LYVE-1-dependent interaction is crucial for the efficient clearance of macrophages from the injured heart to the mediastinal lymph nodes, thereby resolving inflammation and improving cardiac function (70, 173). Notably, during neonatal heart regeneration, LYVE-1-mediated macrophage retention within the myocardium supports tissue repair, revealing a context-dependent role for this receptor (57).

Lymphatic endothelial cells also produce CCL2 (MCP-1), which exerts dual functions in cardiac repair following myocardial infarction (189). LEC-derived CCL2 not only induces macrophage chemotaxis but also activates the AKT/ETS1 signaling pathway in an autocrine manner, thereby enhancing VEGFC expression and promoting lymphangiogenesis (189). Conditional knockout of the Ccl2 gene in LECs exacerbates cardiac dysfunction, impedes lymphangiogenesis, and impairs macrophage clearance via cardiac afferent lymphatic vessels, underscoring the critical role of LEC-expressed CCL2 in coordinating lymphatic expansion and immune cell trafficking (189). Studies have also shown that CCL2 deficiency reduces macrophage infiltration, delays phagocytosis, and attenuates left ventricular dilation after myocardial infarction (190, 191).

Furthermore, intercellular adhesion molecule-1 (ICAM-1) on LECs actively participates in leukocyte recruitment and immune microenvironment modulation (192). In response to inflammatory cytokines such as TNF-α, LECs upregulate ICAM-1 expression, which mediates dendritic cell adhesion and trans-lymphatic migration (193). Single-cell transcriptomic and spatial analyses have revealed that LEC-specific ICAM-1 deletion results in exacerbated inflammation and fibrosis following myocardial infarction, along with an accumulation of pro-inflammatory neutrophil and macrophage subsets (192). This defect is attributed to impaired immune cell drainage via LEC-specific ICAM-1 signaling, demonstrating that ICAM-1 facilitates leukocyte transendothelial migration into lymphatic vessels (192).

At the level of immunomodulation, cardiac lymphatic vessels exhibit dynamic functions. Initially, they actively recruit immune cells via secretion of chemokines like CCL21. In the later post-injury phase, lymphatic endothelial cells undergo transcriptional reprogramming, mediated by factors such as the transcription factor Tbx1 (178, 194). This leads to the upregulation of molecules including CCL21 and ICAM1 (178). This reprogramming enables lymphatics to specifically recruit immune-suppressive cell types, such as tolerogenic DCs and regulatory T cells (Tregs) (195), while concurrently inhibiting the activation and expansion of autoreactive CD8^+^ T cells.

Ultimately, at the level of establishing immune tolerance, this lymphatic-orchestrated microenvironment ensures that suppressive immune cells and appropriately presented antigen are drained to the lymph nodes. This process further promotes the active resolution of inflammation and curtails excessive autoimmune responses, thereby creating favorable conditions for cardiac repair.

## 5 Discussion

The pathogenesis and progression of heart failure involve a complex interplay of hemodynamic, neurohormonal, and inflammatory perturbations. While contemporary therapies targeting these axes have markedly improved clinical outcomes, a significant proportion of patients fail to achieve complete cardiac reverse remodeling, often exhibiting residual myocardial edema and fibrosis. This persistent pathology underscores the limitations of conventional strategies, which primarily focus on reducing fluid production or modulating inflammatory signals without actively enhancing their clearance from the myocardial interstitium. The evidence synthesized in this review posits the cardiac lymphatic system as a crucial, yet historically underexplored, physiological pathway that may hold the key to overcoming this therapeutic impasse. By orchestrating fluid homeostasis and immune surveillance, a functional lymphatic network is intrinsically linked to the processes of repair and regeneration, positioning its therapeutic augmentation as a compelling novel strategy to promote sustainable reverse remodeling (139).

The cardinal function of the lymphatic system in mitigating myocardial edema provides a direct mechanistic link to structural and functional recovery. As detailed, impaired lymphatic drainage leads to interstitial fluid accumulation, increasing wall stiffness, impairing coronary perfusion, and promoting a pro-fibrotic milieu. Conversely, effective lymphatics, particularly when therapeutically stimulated during the acute and subacute phases of injury, facilitate the resolution of edema. This alleviates mechanical stress on cardiomyocytes and the extracellular matrix, creating a permissive environment for the recovery of contractile function and the initiation of reverse remodeling. Importantly, this role extends beyond passive drainage. The system’s reliance on cardiac motion for lymph propulsion establishes a fascinating feedback loop whereby improving systolic function concurrently enhances lymphatic efficiency, potentially creating a virtuous cycle of recovery. This interdependence highlights that therapeutic strategies aimed at reverse remodeling may benefit from co-optimizing both pump function and drainage capacity.

Equally critical is the lymphatic system’s sophisticated role in immune modulation, which operates in concert with its fluid-clearance function. Myocardial injury triggers an inflammatory cascade essential for debris clearance but detrimental if unchecked. The lymphatic vessels serve as the principal route for egress of antigen-presenting cells, neutrophils, and lymphocytes from the heart to draining lymph nodes. This regulated trafficking is not a passive process but is actively governed by molecular cues such as CCL21-CCR7 and S1P-S1PR1 gradients. By clearing activated immune cells and pro-inflammatory cytokines, the lymphatics facilitate the timely transition from the inflammatory phase to the reparative phase (196). Failure in this clearance mechanism, as observed in chronic heart failure or with aging, results in the persistent low-grade inflammation that fuels ongoing fibrosis and adverse remodeling. Therefore, enhancing lymphatic function represents a strategy to fundamentally resolve, rather than merely suppress, detrimental inflammatory responses, thereby addressing a root cause of incomplete remodeling.

The molecular architecture governing lymphatic development and response offers a rich repository of druggable targets to achieve therapeutic lymphangiogenesis. The VEGF-C/VEGFR-3 axis stands as the master regulator, and its targeted delivery has consistently shown promise in preclinical models of myocardial infarction and pressure overload, improving drainage, reducing inflammation and fibrosis, and enhancing function (128). The complexity of this pathway, involving essential co-factors like CCBE1 and the protease ADAMTS3 for VEGF-C maturation, presents opportunities for precise therapeutic intervention. Beyond growth factors, transcriptional regulators such as PROX1 and FOXC2 integrate mechanical and biochemical signals to maintain LEC identity and valve integrity. Modulating these regulators or their upstream mechanosensors, like integrin complexes, could enhance lymphatic adaptability to pathological stresses. Furthermore, the stromal interface, exemplified by the LYVE-1-HA axis, and immune trafficking molecules like CCL21 provide additional levers to fine-tune lymphatic growth and immunomodulatory capacity specifically within the cardiac context. Collectively, these molecular components form a robust foundation for therapeutic targeting, where the emerging application of bioactive peptides and small-molecule drugs holds promise for achieving precise spatiotemporal control of lymphangiogenesis and optimizing cardiac repair strategies (197–201).

Despite this promising outlook, significant translational gaps must be bridged to move from mechanistic insight to clinical application. A primary challenge is the optimal timing of intervention. While augmenting lymphangiogenesis is beneficial acutely, its effects in the chronic, fibrotic heart require further elucidation (139). The potential heterogeneity of LEC origins and the organ-specific characteristics of cardiac lymphatics also demand careful study to avoid off-target effects (202). Moreover, the impact of pervasive comorbid conditions such as diabetes, atherosclerosis, and aging—all of which impair lymphatic structure and function—cannot be overstated. Any effective therapy will likely need to overcome these baseline deficits. Future research must prioritize the development of cardiac-specific delivery systems for lymphangiogenic factors, the discovery of small-molecule agonists of key pathways like VEGFR-3, and a deeper understanding of how lymphatic therapy integrates with established heart failure pharmacotherapies (109, 203). The ultimate goal is a tailored approach that restores the lymphatic system’s homeostatic capacity, complementing existing treatments to achieve more complete and durable reverse remodeling.

## 6 Conclusion

In conclusion, the cardiac lymphatic system emerges not as a mere bystander but as an active, indispensable participant in myocardial recovery. Its dual mandate of fluid clearance and immune regulation addresses fundamental shortcomings in current heart failure management. The evolving understanding of its developmental signals, molecular regulators, and pathophysiological alterations provides a robust scientific foundation for targeting this system. Harnessing the lymphatic system’s potential represents a paradigm shift, aiming to reconstitute the heart’s intrinsic milieu for repair. As research progresses to refine therapeutic strategies and validate their efficacy in clinical settings, augmenting cardiac lymphatic function holds substantial promise for unlocking new frontiers in the pursuit of complete and sustained reverse remodeling in heart failure.
